# The MSc Psychiatry at Cardiff University: Introduction of New Modules Further Supporting Continuing Professional Development in Psychiatry

**DOI:** 10.1192/bjo.2022.129

**Published:** 2022-06-20

**Authors:** Athanasios Hassoulas

**Affiliations:** Cardiff University School of Medicine, Cardiff, United Kingdom

## Abstract

**Aims:**

The *MSc Psychiatry at Cardiff University* is an established postgraduate programme offering students a sound theoretical basis in psychiatry as a medical science and specialty. The programme currently offers six taught modules (focusing on mood and anxiety disorders, psychosis, old age psychiatry, forensic psychiatry, substance misuse, and child and adolescent psychiatry), as well as a dissertation module that students complete towards the end of the programme. In catering for the professional needs of clinical students and students pursuing careers in academia, two additional taught modules have been proposed exploring *Leadership and Management in Psychiatr*y and *Advances in Psychiatric Research*. Feedback on the proposed introduction of the new modules was collated from the current full-time and part-time student cohorts.

**Methods:**

A total of 57 students currently enrolled on the programme were surveyed in relation to the proposed additional taught modules. The survey was created using Microsoft Forms and deployed via the programme's virtual learning environment (i.e., Blackboard). A mixed methods design was employed, with both Likert scale and open-ended items included in the survey. Students were informed that future cohorts would be offered a choice between the existing *Forensic Psychiatry & Substance Misuse* module and the proposed *Leadership and Management in Psychiatry* module, as well as a choice between the existing *Child and Adolescent Disorders* module and the proposed *Advances in Psychiatric Research* module.

**Results:**

Responses from the current student body were collated and analysed. A total of 29 (51%) students surveyed were medical professionals, with the remaining 28 (49%) students being science graduates or other clinical professionals. Descriptive analysis of the quantitative data revealed that an overwhelming majority of students viewed the introduction of the new modules as a positive development that would further enhance the student learning experience and continuing professional development. Content analysis of the qualitative data revealed further insights on the nature of the proposed modules and preferences on how these should be included within the existing programme schedule.

**Conclusion:**

Students currently enrolled on the *MSc Psychiatry* favour the introduction of the proposed modules tailored to support professional development. Specifically, students view the proposed module on *Leadership and Management in Psychiatry* as catering to the needs of clinicians working in a variety of healthcare settings, whilst the proposed module exploring *Advances in Psychiatric Research* was considered to supplement existing course content on evidence-based medicine and caters for students with an interest in pursuing a career in academia.

